# Evaluation of Arts based Courses within a UK Recovery College for People with Mental Health Challenges

**DOI:** 10.3390/ijerph15061170

**Published:** 2018-06-04

**Authors:** Joanna Stevens, Catherine Butterfield, Adrian Whittington, Sue Holttum

**Affiliations:** 1 Aldrington House, Sussex Partnership NHS Foundation Trust, 35 New Church Road, Hove BN3 4AG, UK; catherine.butterfield@sussexpartnership.nhs.uk (C.B.); adrian.whittington@sussexpartnership.nhs.uk (A.W.); 2 Salomons Centre for Applied Psychology, Canterbury Christ Church University, Tunbridge Wells TN1 2YG, UK; sue.holttum@canterbury.ac.uk

**Keywords:** arts, mental health, personal recovery, recovery college

## Abstract

No previous studies have evaluated arts based recovery college courses. Yet arts may assist in personal recovery, as often defined by service users, through social connection and personal meaning. This interdisciplinary study evaluated (i) whether self-reported wellbeing and arts activities increased following arts based recovery college courses, and (ii) how students, peer trainers and artist-trainers understood courses’ impact. The design was mixed-methods. Of 42 service user students enrolling, 39 completed a course and 37 consented to provide data. Of these, 14 completed pre and post course questionnaires on mental wellbeing and 28 on arts participation. Post course focus groups were held with six of eight peer trainers and five of seven artist-trainers, and 28 students gave written feedback. Twenty-four students were interviewed up to three times in the subsequent nine months. There were statistically significant increases in self-reported mental wellbeing and range of arts activities following course attendance. At follow-up 17 of 24 students reported improved mental wellbeing, while seven reported little or no change. Some spoke of increased social inclusion and continuing to use skills learned in the course to maintain wellbeing. Initial in-course experience of ‘artistic growth’ predicted follow-up reports of improvement. Future controlled studies should employ standardized measures of social inclusion and arts participation.

## 1. Introduction

### 1.1. Arts and Wellbeing

A 2017 UK parliamentary enquiry on Arts, Health and Wellbeing [1] (p. 34) identified, “An expanding body of evidence to support the contention that the arts have an important contribution to make to health and wellbeing.” The inquiry noted positive contributions in a range of areas including “aging, long term conditions, loneliness and mental health” [1] (p. 4), but also the need for further research. In relation to mental health and recovery, research suggests that participatory arts activities can increase people’s sense of hope, meaning and identity [2], and confidence, connectedness and empowerment [2,3]. Some studies have reported that arts activities and their community context were experienced as a welcome change from a mental health service environment and its focus on illness, and that new creativity and an artistic identity could be forged [2,3,4].

### 1.2. Recovery and Recovery Colleges

Anthony’s [5] (p. 15) often cited definition of mental health recovery suggests that it is “a deeply personal unique process”, involving “goals”, and living “a satisfying, hopeful, and contributing life even with limitations caused by illness.” Slade [6] (p. 37) has called symptom-reduction “clinical recovery”, contrasting it with “personal recovery”, consistent with Deegan’s suggestion [7], from her experience as a survivor of unhelpful aspects of the mental health system, of recovering a life. Recovery as defined by service users has been described as “the individual moving towards being able to live the kind of life he or she wants to live” [2] (p. 792).

Consistent with these definitions, a review of qualitative studies of how mental health service users viewed recovery other than symptom-reduction produced five themes: connectedness, hope, identity, meaning and empowerment [8]. The UK Department of Health’s Implementing Recovery through Organizational Change program aimed to support individual recovery as well as establish recovery principles within organizations [9]. Following wide stakeholder consultation, its proposals included providing recovery colleges offering co-produced educational courses.

In theoretical terms, building a meaningful life either outside or alongside mental health treatment could be understood as a life centered on one’s own interests. Interests are theorized [10,11] as one aspect of an instinctive, biologically based system developed from infancy in the context of attachment relationships. Extending the work of Bowlby [12] and Ainsworth [13], Heard et al. [11] posit that our exploratory, interest-sharing lives are developed through early attachments with caregivers, require support throughout life and are integral to overall health. They see caregiving as having two forms; (i) to protect from danger, comfort and regulate arousal and (ii) to nurture interests and develop skills for sharing interests with peers. We suggest this latter interest-nurturing type of caregiving may be enabled through recovery college courses [14].

O’Neill [15] also discusses meaningful engagement in cultural activities, suggesting that people need to meet more than instrumental aims such as health benefits anticipated from exercise. This is consistent with the use of social identity theory [16] to explain evidence that some group-based health interventions are more effective when those participating already have an interest in the focus and it is personally meaningful. We are sustained by belonging to groups with which we identify and that maintain our positive social identity [17]. O’Neill [15] (p. 22) argues that whilst the benefit of short-term arts projects is well recognized, his epidemiological research “suggests that a strategy promoting less intensive attendance at cultural organizations among vulnerable communities may be able to achieve a health impact at a population level.” His review of the literature suggests that “simply going to a museum, art gallery, film or concert on a regular basis increases longevity” [15] (p. 22).

### 1.3. Evidence on Recovery Colleges

The first UK recovery college opened in 2010 in London [18], with nearly 40 others established since including in Norfolk, Nottingham, Scotland, Sussex, Manchester, as well as further afield in Ireland, Australia, Japan and Canada [19]. Narrative descriptions [20,21], psychiatrists’ perspectives [22] and evaluation data from recovery colleges have documented largely positive outcomes. These have included participants’ perspectives on what makes recovery colleges effective [23], their impact and changes in organizational practice [24], factors that support and hinder attendance [25], participants’ experience [26], and financial savings through reductions in service use [18,27].

To date participant numbers in studies remain mostly small, with notable exceptions [23,27]. Few evaluations have followed people up after they have completed a course. In addition, searches of the literature indicated that there were no published studies of arts based recovery college courses. Arts are mentioned in some studies [20,23,28], but not evaluated separately from other recovery college courses.

### 1.4. Service Context

The arts based recovery college courses were provided by an arts-and-health program called Make your Mark (MYM) for people with mental health and related challenges. MYM arose from work undertaken since 2010 to increase access to the arts within Sussex Partnership National Health Service (NHS) Foundation (mental health) Trust. Earlier projects included developing a gallery tour for people with memory problems, their families and carers, based on other work in the USA and UK [15,29,30,31,32,33]. By 2015 MYM, hosted within Sussex Partnership NHS Foundation Trust, and funded through the trust’s charity Heads On, was established with the aim of integrating “creativity into the fabric of health care” [34]. O’Neill’s work [15] underpins the present authors’ aim that arts projects must point beyond themselves and include collaboration with cultural partners. Consultation with service users, NHS staff, arts partners and general public led us to the recovery college model.

### 1.5. Rationale and Aims for the Current Study

Recovery colleges are a relatively recent innovation in mental health services and previous research has been sparse, with many studies small scale and qualitative. Research has also not examined service users’ experiences both in-depth and over time or in relation to arts based recovery college courses. The aim was to use a mixed-methods design [35], both quantitative and quantitative, for immediate evaluation of course experience and outcome, and to supplement this with follow-up interviews up to nine months subsequently, to capture any longer-term benefits people might report in their own words. This could provide a sense of what it might be important to measure in future controlled studies [36].

### 1.6. Research Questions

1. What, if any, changes in wellbeing would be reported on standardized questionnaires by course participants pre to post attendance?
2. Would there be any change in arts participation from pre to post course?
3. What would be the experience of artists, peer trainers and students immediately post course, and for individual students at three month follow-up interview?
4. What, if any, and if so what type of changes in wellbeing would service user students describe at three, six and nine months follow-up?
5. What, if any, other changes related to wellbeing would students report in the follow-up period?
6. Would students report any increase in arts participation in the follow-up period?
7. Would there be any patterns in terms of those who report most or least mental wellbeing improvement at six and nine months follow-up in relation to their three month reported experience of courses?

## 2. Materials and Methods

Seven arts based courses were co-developed, co-produced and co-facilitated by commissioned artists (expert by arts knowledge and practice) and peer trainers (expert by lived experience of mental distress). The courses are briefly described in Table 1.

### 2.1. Design

A mixed-methods design was used. Two standardized wellbeing scales were used to assess self-reported student wellbeing pre and post course, an arts participation measure to capture any change in arts participation, anonymous qualitative comments post course, focus groups with the trainers (artists and peer trainers), and interviews with students at three, six and nine months post course. In relation to seeking possible links between service users’ experiences of MYM courses and subsequent reports of wellbeing, exploratory pattern matching was used [37].

### 2.2 Participants

The eight peer trainers (including a relief trainer) and seven artists involved in course delivery were each invited to take part in a separate artist and peer trainer focus group. The 42 service users who completed courses (attended at least 75% of sessions) were invited to complete pre and post course questionnaires on wellbeing and arts participation, to provide anonymous written feedback on their experience of the course, and to be interviewed three, six and nine months after the courses ended. All 42 service users consented to provide pre and post course data, and 37 to be interviewed at follow-up points. However, due to course attrition, participants not being in attendance for pre and/or post data collection, incomplete questionnaires, or not being available for follow-up interviews, data were incomplete. Information about the number of participants taking part in each component is provided in the Results section.

### 2.3. Measures

As part of routine Sussex Recovery College (SRC) processes, quantitative data were gathered from the students pre and post course. This included wellbeing scales as follows.

#### 2.3.1. Short Warwick-Edinburgh Mental Well-Being Scale [38]

This 7-item scale includes items such as, “I’ve been dealing with problems well” and “I’ve been feeling close to other people”. Answers on a 5-point Likert scale range from “None of the time” to “All of the time”. It has been used to evaluate change in public health interventions, including in populations with severe mental health diagnoses [38,39], and in evaluating recovery colleges [19].

#### 2.3.2. CHoice of Outcome in Cbt for Psychoses (CHOICE) [40] Short Form

This is a service user led measure assessing psychological recovery and mental health following cognitive behavior therapy (CBT) for psychosis [40]. However its items are not specific to CBT or psychosis. Items on which respondents are asked to rate themselves include “Positive ways of relating to people” and “Understanding my experiences (e.g., beliefs, thoughts, voices, and related feelings).” Ratings range from 0 (worst) to 10 (best). Item 12 is a personal goal the respondent may set for him or herself and rate in the same way and was not used in this evaluation. The short form has been used as an outcome measure for Sussex Recovery College since the College’s inception. The original form of CHOICE was reported to have good psychometric properties [40] but no information was available on the shorter version.

#### 2.3.3. Arts Participation

This was measured using a tool designed in consultation with service users to be easy to use and to reflect that service users can sometimes experience difficulties leaving their home. It comprises one landscape page divided into four quadrants. The first quadrant presents 13 arts activities written in bubbles so that the participant can tick any applicable to them for the previous three months (including “Crafts”, “Singing”, ”Going to galleries or museums”, and “Painting”). The second quadrant presents eight locations (including “At home”, “Library”, “TV or radio”, and “Attend a group or class”). The third quadrant presents seven different people or groups with whom participants may have engaged in an activity (including “With pets”, “Family”, and “On my own”), and the fourth quadrant contains the question, “How often have you taken part in creative activities?” and presents five ratings of frequency scored from 1 (once) to 5 (daily). Only face and content validity were established for this measure.

### 2.4. Qualitative Data

Student post course qualitative data were taken from anonymous course feedback forms (Supplementary Table S1). Students were asked to rate nine positive statements on a scale of 1 to 5 (1 = strong agreement and 5 = strong disagreement). They were invited to comment if they wished. For example, being asked to rate the statement “I found this course helpful and informative” was followed by “What was most helpful/informative for you (if relevant)?” Rating the statement “I found it helpful that the course included facilitation by a Peer Trainer with lived experience” was followed by “What was the most helpful aspect of this (if relevant)?” They were asked to rate the helpfulness of the tutors, helpfulness of other students for their learning, the materials and the venue (Table S1). They were additionally asked to provide any further comments. Only the qualitative data are reported here. The ratings can be found in Table S1. The artists’ and peer trainers’ experience of facilitating courses was assessed through separate focus groups each lasting one hour and co-facilitated by the second author and a peer expert by experience. Topics were about their experience overall, of the venues, of working with the students, of working with their co-facilitator, of any impact on themselves, whether they would do anything differently, and anything else that had not been covered.

The interviews at follow-up were semi-structured (Tables S2–S4). Students were asked how their arts participation had been since the end of the course (three month follow-up) or since the previous follow-up (six and nine month interviews), and how they felt their health was (physical and mental wellbeing). At three month follow-up participants were also asked to give feedback on the course, its content, the tutors and venue.

### 2.5. Procedure and Analyses

Clinical governance approval was provided by Sussex Partnership NHS Foundation Trust prior to starting (Figure S1). The research assistant (second author) visited all courses to explain the evaluation and seek informed consent. The focus groups took place within a week of the course graduation ceremony, approximately one month after the end of the courses. The peer focus group took place at a trust property, and the artist focus group in an arts venue. The focus groups took the form of semi structured interview and were recorded and transcribed verbatim, as were all recorded follow-up interviews.

Paired *t*-tests were used to test for change on the two standardized wellbeing scales, and Wilcoxon signed ranks test for the unstandardized arts participation measure. Significance level was set at 0.05 (two-tailed) for all quantitative tests. They were carried out using Statistical Package for the Social Sciences, version 23.

Inductive thematic analysis [41] was used for all qualitative data analysis except identifying themes of improved mental wellbeing, lack of change in wellbeing, and data indicating doing more or less arts activities. Deductive analysis was used in that the authors sought data conforming to these *a priori* themes in follow-up interview data. Initial coding of transcripts and course feedback were undertaken by the second author, who also recorded and transcribed the majority of interviews. Due to timing the six month interviews were transcribed by an outside person who signed a confidentiality agreement.

Recordings were listened to several times and transcripts read and re-read. For each data set, codes were independently identified. Initially the focus groups and course feedback data were transcribed and coded. The second author initially coded and reviewed the data. Themes not relevant to the research were excluded (for example stories participants told about how they came to mental health services). An artist (who was expert by experience, had been involved in setting up the project and had co-run the focus groups) and the project coordinator met with the second author to review and agree themes. At this stage each theme was debated and honed. The process of data immersion, transcribing, coding and reviewing was repeated for the service user interview data sets.

The second author then met the first author and a further expert-by-experience artist uninvolved in the project, to review and agree themes against the data. Further coding also happened at this stage where different people identified different themes or saw existing themes in a different light. For example, an initial code of personal growth was later incorporated into *improved mental wellbeing*. Further coding and discussions were done with all authors.

In order to provide a descriptive illustration of which participants described most and least improvement in wellbeing at different follow-up interviews, the authors classified participants into four groups: ‘Sustained improvers’ were those who self-reported improvement at more than one of the three follow-ups. They were only included in this group if any report of no change was only at first follow-up and subsequent reports were of improvement only. To qualify as ‘improvers’, participants had to have reported improvement on at least one follow-up, and any report of no change had to be followed by only reported improvement on a subsequent follow-up. To be designated ‘possible improvers’, participants’ report of improvement was followed by report of no change. To be designated ‘no change’ they did not report improvement at any follow-up, and reported no change at more than one follow-up. For pattern matching, because of small numbers, Fisher’s exact test of significance was used to assess whether students for whom specific themes were identified at three month follow-up concerning their experience of courses were associated with membership of (a) the sustained improvers or improvers, versus (b) the possible improvers or no change group.

#### Quality Criteria

After the initial round of coding, the second author’s coding was checked by people with diverse perspectives as already described. The fourth author, who was independent from the setting up or running of the project, offered monthly research supervision to the second author, who was employed to evaluate the project, and who used this time for discussion of practical issues and reflecting on potential imposition of personal biases on data coding. All contributors were alert to negative cases, that is, examples of self-reported experience that seemed at odds with majority-reported experience [42]. This was important to avoid under-representing experience that might be unanticipated but could be important.

## 3. Results

### 3.1. Wellbeing and Arts Participation Pre and Post Course

Of the 42 service users completing courses, 41 were in treatment for moderate to severe mental health issues in secondary or higher level care (including low-secure forensic care and supported housing). One did not provide this information. Fourteen of the 42 participants provided full pre and post data for the two standardized measures and 28 for arts participation. Table 2 shows that students reported significantly improved wellbeing on both standardized measures after compared to before their course attendance.

In terms of arts activities, students collectively reported significant increases on only one indicator (number of different activities) on the arts participation questionnaire, when these were corrected for their course-attendance (Table 3).

### 3.2. Post Course Perceptions of Course Experience by Artists, Peer Trainers and Students

Anonymous written post course feedback was provided by 28 participants. The peer evaluation focus group consisted of six of the eight peer trainers including the relief trainer, who had been involved in setting up the courses and assigning the peer trainers to their positions within the program. The artists’ evaluation focus group included five of seven artists. Written feedback was received from the remaining two. For the longitudinal evaluation involving interviews three, six and nine months after the courses ended, 37 students provided informed consent, and 22 provided data at first follow-up, including at least one student from each course. There were 12 women and 10 men (age range 28–82 years). Interview lengths ranged from 12 min to 1 hour and 5 min.

The themes from the focus groups with artists and peer trainers, anonymous written feedback from students and three month follow-up interviews are summarized in Table 4, Table 5 and Table 6, with overarching theme names of *Transforming students’ lives, Development as trainers* and *Overcoming hurdles*.

#### 3.2.1. Improved Mental Wellbeing

Participants of both focus groups (artists and peer trainers), written feedback from students, and participants at three month follow-up interview mentioned students’ improved mental wellbeing. This included reduced distress and increased confidence and self-worth:

*“It really improved their mental health and their anxiety levels had gone down, and one was quite suicidal at the beginning but by the end she was in much more positive space.”*

—Lindy, artist (all names are pseudonyms)

*“It was a wonderful course and I really want to do more. I felt very suicidal before and now I have remembered a part of me I had forgotten so thank you.”*

—Student (anonymous feedback)

*“From turning up and being hunch-shouldered, really unable to talk to, literally she, you saw at the graduation she was walking with her head up. She walks with her head up and chest out now.”*

—Michael, artist.

*“I think it was quite powerful how helpful it was with confidence”*

—P32, 3m (Participant 32 at three month follow-up)

*“It was a big lift of self-esteem because obviously doing something like that, in a place like that [museum] obviously you do feel that you have more value.”*

—P8, 3m

#### 3.2.2. No Change in Mental Health

Some participants interviewed at three month follow-up had felt benefit while on the course but could be viewed as negative cases in relation to this theme, in that they felt that this had not been maintained very well or at all afterwards:

*“It’d be better if I did what I was doing on the course, I was a lot—my voice was a lot better.”*

—P36, 3m

One participant felt he might have benefited more if he had attended from the beginning:

*“If I’d of come from the beginning as I say from the first session to the last obviously and been more acquainted with the people there and that…”*

—P16, 3m

#### 3.2.3. Artistic Growth

A sense of *artistic growth* seemed to be evident in all three groups and was discussed at three month follow-up. Several participants spoke at interview about aspects of courses that they experienced as inspirational, or having experienced the course as liberating, enabling people to feel positive about themselves and their artistic capabilities, motivated, and able to express feelings in new ways:

*“I have developed an artistic personality.”*

—Student

*“The creativity of the students that they came up with their own story to tell […] they blew us out the water.”*

—Sindy, peer trainer

*“And learning that things don’t have to be perfect, because I found that really –‘Oh no! We haven’t got to make a noise the same’ that was really hard, because I get stuck in rules and regulations and things. So that was quite eye opening.”*

—P32, 3m

*“I danced this emotion. I’d had this therapy session and there was all this anger.”*

—P11, 3m

Some students mentioned motivation to use their new skills at home:

*“I can take things home and I wanted to actually do things at home. I wanted to actually do what I could.”*

—P3, 3m

Some compared arts based courses positively with mental-health-focused ones:

*“I think it gives people a lot of freedom as well. So they’ve got that two hours to do what you want and you don’t get that in sort of health related courses. I would say it’s just as valuable as a ‘living with bipolar’ course”*

—P10, 3m

#### 3.2.4. Inspiring Venue

For all groups, the nature of the community-based venue seemed to add something special to their experience. A number of participants talked at follow-up about the *inspiring venue*. This could be about the nature of the venue itself, or that it was not a mental health service location, or a combination of these:

*“The venue was amazing—that adds something to it. We had this studio, a proper studio on the side of the De la Warr Pavillion which is an incredible building in itself.”*

—Liam: Peer trainer

*“It’s a great bonus that the course is being held at Brighton Museum as it is both quiet and inspirational.”*

—Student

*“That was the most phenomenal thing. It felt so special, privileged to be [at museum] […] it’s like living there really, because you’re there for that amount of time and you’re allowed to be there. That was lovely.”*

—P8, 3m

*“I preferred it a lot, I thought it was more relaxed and informal. They can be hard to go into, sort of [NHS] trust places when you’re not feeling good, just sort of triggering memories sometimes. I thought it was really good to be away from the Trust.”*

—P32, 3m

#### 3.2.5. Learning Skills

Some students self-reported, and peers saw them as developing and using skills for coping with difficulties as a result of attending a course:

*“They’d report on experiences where they’d been able to cope with some sort of difficulty during the week as a direct result of learning something on that course.”*

—Liam, peer trainer

[What was helpful:] *“Learning about breathing exercises to help make different sounds and also to help deal with stress.”*

—Student

*“Discovering other skills which I perhaps didn’t know I had, or hadn’t had whatever reasons to explore it, […]. This is something which I’m just opening myself up to.”*

—P3, 3m

#### 3.2.6. Positive Risk-Taking

Peer trainers and students gave feedback suggesting that students had been spurred to do things they would not have done otherwise. Several participants also talked at interview about feeling able to go outside their comfort zone, which seemed best summed up as *positive risk-taking*. Several people conveyed that they felt anxious at first, but then were able to relax and participate:

*“One of the students who hadn’t participated in the dance very much was really into the dance. I was so pleased to see that. Cos she was, she had been, it was very much out of her comfort zone.”*

—Nina, peer trainer

*“I feel this helped my confidence but was initially quite anxiety-provoking. The course felt nice and relaxed and the trainers were lovely.”*

—Student

*“I could see everyone else was making an effort and things—it sort of pushed me to go out beyond my comfort zone. I never thought I’d be doing like the dance bit or anything. I’ve never been able- I never had the confidence to do it but yeah.”*

—P13, 3m

*“So working with a group of people in its own right was a very difficult thing to do, but when you’re doing something fun it makes it a little bit easier.”*

—P19, 3m

One student appreciated having higher expectations placed upon her capability than usual:

*“It was almost like a nice surprise to actually have a higher expectation as opposed to the norm, which may have a lower expectation of people with chronic illness, mental health issues.”*

—P3, 3m

#### 3.2.7. Positivity of Social Aspects

Immediately post course this theme only seemed present in the student feedback but it was prominent there. For example in response to the question as to rating whether the course had helped in their recovery, one student wrote:

*“Being in medium sized group social interaction. Applying myself and becoming more aware of people’s situations, empathy.”*

—Student

Some students also talked about the positive social aspect at interview. As with *positive risk-taking*, there was a sense of pushing beyond initial difficulties:

*“It was nicer having all the people around because you can look at what everyone’s doing, everyone’s having a good laugh, and everyone really sort of helped each other out […] so that kind of class with extra people round me making noise didn’t matter at all, in fact it helped I think, me enjoy it more.”*

—P5, 3m

*“I find interacting with people difficult. […] The Recovery College for me just completely removed a lot of those difficulties [while attending the course]. […] To randomly walk into a room with a load of strangers and feel comfortable, that’s not something that I’d normally feel comfortable with.”*

—P19, 3m

*“Having something to do meant people could distract the attention away from themselves, focus on something than just themselves. And we bonded as a group, everyone formed bonds with each other. It was very good.”*

—P28, 3m

#### 3.2.8. Tutors Special

A few people mentioned the *tutors* and feeling that they brought something *special* to the course, both through their perceived dedication and their supportive stance:

*“That’s [artist travelling a long way] real commitment, which kind of inspired a lot of people on the course to show a similar level of commitment to it as well.”*

—P10, 3m

*“They [tutors] were lovely, very supportive so I didn’t feel – although I’m quite anxious about missing things and then going back, I felt really supported and I wanted to go.”*

—P11, 3m

### 3.3. Development as Trainers

The second main theme was *Development as trainers*. Its sub-themes are presented in Table 5.

#### 3.3.1. Practice Development

Both artists and peer trainers voiced a sense of developing their practice:

*“It was for me the first time that for a project I’ve mixed art, movement and nature together into something coherent and it was wonderful to go, ‘Oh this works!’ So professionally, that was something I’d like to build on. That was great!”*

—Hatty, artist

*“I started painting at home again. And I’m doing commissions again now.”*

—Sindy, Peer trainer

#### 3.3.2. Personal Confidence and Self-Esteem

Artists and peer trainers both talked in the focus groups about increased confidence and self-esteem:

*“It’s been a really rich experience, which is always really good for the self-esteem.”*

—Lindy, artist

*“How it’s impacted on me? For me I think it is: ‘I can do that, I can do what- I can go off and teach mono-printing or whatever.’ […] I feel more confident, yeah more confident in doing that.”*

—Martin, peer trainer

#### 3.3.3. Professional Pride

Only artists talked about something that seemed like professional pride, which seems to go beyond developing practice and confidence:

*“I was able to talk about it at a conference—the [name of conference]. I felt really good about it. It was part of our portfolio so that felt really, really nice.”*

—Pamela, artist

*“You know, people say, “What are you up to?” and this is one of the things I’m up to. I’m feeling quite proud.”*

—Hatty, artist

#### 3.3.4. Positive Risk-Taking

Peer trainers but not artists espoused the sub-theme of positive risk-taking immediately post course. It was similar to the sub-theme of the same name as applied to students (see Section 3.2.6):

*“I was well outside my comfort zone but in a safe space, so it was OK.”*

—Lorraine

*“I would not have believed that I would come out of this so enthusiastically when I started it because I was completely out of my comfort zone.”*

—Liam

### 3.4. Overcoming Hurdles

#### 3.4.1. Appreciation of Support and Supervision

Both artists and peer trainers talked about appreciating support and supervision. For one artist, supervision seemed to help her accept how things were, as opposed to perhaps some expectations about greater attendance or easier engagement for some students:

*“I’d had a supervision and it was about particularly two people, […] who were quite fragile I would say […]. That following week it was just those two individuals, so […] we could just really go with their rhythm. I had a bit of a penny drop moment where I just thought, ‘It’s just this is what it is, it’s just this.’ You know just this experience, just being that kinda intimacy we built with the small number was great.”*

—Hatty, artist

*“Because we had induction […]. We had the peers and the trainers together and I think that day for me was amazing. And a lot of learning from all sorts of different people—the amount of knowledge in that room and the amount of creativity and everything else.”*

—Lorraine, peer trainer

#### 3.4.2. Concern over Attendance

Peer trainers and artists alike sometimes voiced concerns or having had expectations that attendance might have been greater:

*“It just seemed a shame it was just ‘OK these are three people—we can’t get any more on there.’”*

—Michael, artist

*“I don’t know whether people were put off because they didn’t know [the venue] and perhaps didn’t come.”*

—Tana, peer trainer

#### 3.4.3. Co-production: The good, the Bad and the Ugly

There were times when co-production felt both rewarding and challenging for both artists and peer trainers:

*“It didn’t feel separate when we were doing it. We had our own roles but it was cohesive.”*

—Sindy, peer trainer

*“It didn’t feel like it was an even sort of relationship for me. It was more like a facilitator and an assistant.”*

—Lindy, artist

*“I felt that there was an assumption that we were non-disabled and didn’t have experience of mental health histories and that they did.”*

—Pamela, artist

#### 3.4.4. Meeting and not Meeting Expectations

As alluded to earlier, sometimes the artists’ or peer trainers’ expectations were not met, or they wondered if students’ expectations were not met:

*“I think we lost a couple of people at the outset because it wasn’t what they were expecting to do.”*

—Liam, peer trainer

*“I’ve given [the students] all sketchbooks but they’re not filling a 100 pages a week.”*

—Hatty, artist

#### 3.4.5. Coping with Difference

The range of perspectives among people attending courses was seen as potentially challenging but actually something very positive by both students and peer trainers:

*“Good varied people wide range of experience and diagnosis I suppose, various difficulties; but they just came together and the dynamic quickly settled down.”*

—Liam, peer trainer

*“Considering our very different backgrounds and diagnoses we prioritized the task in hand effectively.”*

—Student

### 3.5. Self-Reported Wellbeing at Follow-Ups

Table 7 shows which participants were designated sustained improvers, improvers, possible improvers and no change (Methods Section 2.5). At six month follow-up 20 students provided data (10 male and 10 female), at least one from each course (age range 26–82). Interview lengths ranged from 9 to 37 min. At nine month follow up 19 students took part. No student from *Stories of a Journey: Recovery on Film* was included at this point. There were 10 female and nine male participants (age range 26–82). Interview lengths were between 4 and 38 min.

Participants’ self-report of improvement or lack of change are exemplified by the quotes in Table 8. They illustrate that several participants who made sustained improvement talked about a course as helping them build confidence to do other things, which could be other arts based activities alone or with others, or social activities.

### 3.6. Other Self-Reported Changes at Follow-Up

Table 9 shows other themes from the three follow-ups. Participants appeared to be *maintaining wellbeing* in a number of ways. The first sub-theme, *using skills learned*, refers to how participants talked about using skills they had learned during the course to cope with difficult feelings and states:

*“Those times when otherwise I might have been a bit frantic and upset and bored, and alone, and now I’ve just gone, ‘I’m going to pick up my art stuff and see what comes out.’ So it’s definitely helped that.”*

—P5, 3m

*“Whenever I get to a bad week I do think back and look back at my study book and I will look through that and it will make me feel a bit better […]. We had like a sketch book.”*

—P14, 6m

*“I can be writing then the next thing I find is that the anxiety is not being held, so I grab the [musical instrument] and the sound of that, it doesn’t exactly help but it does—it allows me to express musically how I’m feeling.”*

—P17, 6m

*“The breathing exercises that I learnt on the Creative Singing for Wellbeing course have been really helpful as taking time to concentrate on breathing and slowing down and becoming more grounded is a very useful tool.”*

—P32, 9m

*“You know where the course is held? It’s given me the confidence to—I like walking around the woods there a few times a week. It sounds silly but I get quite anxious when I’m out […] so it’s helped me. I think I’ve been doing more walking round there and things and taking more attention to what’s around me and the environment.”*

—P13, 3m

Some people talked about doing a *course* other than one in the recovery college, becoming a *volunteer* worker, or obtaining paid *work*:

*“So I’ve done bits of art through that stuff but never had a chance to do, like, proper, proper big courses and things for you to go on, learn some amazing bits—so that course [MYM] was really amazing for me. It actually made me go and do an art and design course with [name of organization], which I’ve just started […] it’s Level 1 City and Guilds.”*

—P5, 3m

Interviewer: *“Would you say that the course helped you? Was that any part of you getting a new job?”*

P14: *“Yeah, it gave me the confidence. It really helped with my confidence because I felt that it had pretty much disappeared and I wasn’t myself anymore and by going to those courses, it really did help.”*

—P14, 6m

*“Completing the course gave me the confidence to apply for an art foundation at [name] college. I was quite convinced that I wouldn’t get a place. I rang them from hospital, the morning of a [medical] procedure. To my great surprise they said they loved my ‘diverse portfolio’ and offered me a place.”*

—P4, 3m

Several participants talked about enriched social lives, which was summarized as *increased social inclusion*, as separate from involvement in work, voluntary work or education and training:

*“It definitely helped me socially. Definitely helped me. I’ve started to get more in touch with my friends in [town] go out on my own as well.”*

—P10, 3m

*“I belong to about four writing groups here in [town], which is really interesting. That saves my life.”*

—P35, 3m

*“I meet up with [friend made on the course] quite a lot as well sort of, that’s helped me.”*

—P13, 9m

### 3.7. Arts Participation at Follow-Ups

Table 10 shows the number of participants who said they were doing more arts activities at six and nine month follow-ups (at three months they could have done an additional MYM course in the interim). Also shown are barriers that participants felt prevented them from doing more arts activities.

Several participants talked about *doing more arts* activities than previously, while others talked about *continuing* previous activities or having them *rekindled*:

*“I’ve bought a calligraphy set. I used to do that years ago and I want to try and get back into that.”*

—P32, 6m

*“I’ve been doing coloring and writing as well […]. I do a bit more now […] [The course] tapped into my creative side. Although its only little, it’s gone up a bit.”*

—P14, 9m

*“It definitely renewed my appetite for creativity.”*

—P8, 9m

Many participants talked about wanting to engage more in arts activities and mentioned different barriers to doing so. These included *competing demands*, *no opportunity*, *physical health* and *mental health*:

*“And I’ve been helping my mum do her packing, when she moves. […] It still hasn’t actually happened. I’ve not been doing much else really.”*

—P44, 6m

*“The people I used to do music with are not around to do music with. So that’s why I’ve not been doing any music really.”*

—P19, 6m

*“And I’m actually half dead. Sort of you know, I’m not completely functioning well.”*

—P35, 6m

*“Recently I tried to join an art group [name]. I joined actually for half a year. It’s all older retired people. I even got extreme anxiety when I went to their sessions because it was quite a big group.”*

—P8, 9m

### 3.8. Patterns in Relation to Most and Least Subjective Improvement

For this analysis, the authors classified participants into two groups. The first comprised all who reported improved mental wellbeing at both the six month and nine month follow-ups, or reported improvement at the latest follow-up (after the first) for which they provided data (see Table 7). Those with no data at both six and nine months were excluded from this analysis. The second group comprised participants who reported no change at both six and nine month follow-ups. One participant was excluded as there were no data. Table 11 shows the associations between belonging in either of these two groups and other self-reported factors from the first follow-up period. There was one statistically significant association and two trends in the seven tests. The finding for *Artistic growth* (see Section 3.2.3 for examples of this) indicates that this, when voiced by students at the first follow-up, predicted later self-reported improvement in mental wellbeing.

## 4. Discussion

### 4.1. Summary of Findings

There was significant improvement on both of the measures of mental wellbeing and on one arts participation subscale. There were qualitative themes that the courses improved service users’ *mental wellbeing* and promoted *artistic growth*. They appeared to *learn skills* and engage in *positive risk-taking*. The *positivity of social aspects* of courses was important for many service users. Peer trainers and artists reported *increased confidence* and *self-esteem* from delivering courses. They *appreciated support and supervision*, which they felt helped them in *overcoming hurdles*.

Based on the follow-up interviews the authors classified 17 participants as reporting improved mental wellbeing, and seven as possibly improved or reporting no change. Several students reported *using skills* learned on their course to *maintain wellbeing*. Many felt more *socially included*. There appeared to be no particular relationship between students’ *prior arts engagement* and subsequently reported benefit. However, if they had talked immediately post course about experiencing *artistic growth*, they were more likely to report *improved mental wellbeing* at later follow-ups. Having experienced *positivity of social aspects* of the course and the *venue as inspiring* showed non-significant trends of association with subsequent reporting of *improved wellbeing*.

### 4.2. Discussion of Findings in Relation to the Literature

Improvement on the two standardized wellbeing scales was encouraging. The improvement on the CHOICE questionnaire short form, which only assesses severity, was comparable to that reported for participants on this subscale following CBT for psychosis [40]. The median number of self-reported arts activities increased significantly following course attendance. However, the number of locations, people or groups with whom these were carried out, and frequency did not change significantly. This could be partly because attending a course is in itself challenging, as has been found in other studies [3,25], and indeed a number of participants alluded to the stress of meeting new people. Recovery course attendance was not included in this measure so as not to falsely inflate arts involvement. Social isolation is a common experience for people labelled with mental health diagnoses, and both social identity theory and evidence suggest a contribution from the negative effects of belonging to a stigmatized social group [43,44]. At recovery college courses, participants may have more easily anticipated being accepted compared to mainstream groups, as suggested by barriers participants mentioned, such as lack of access to former arts related acquaintances, and anxiety attending a large mainstream group.

Nevertheless, participants reported *artistic growth*, entailing not only feeling liberated and opening up during the course, but feeling inspired to take art-forms into their day-to-day life. The theme of *learning skills* was closely related to this, in encapsulating the use people made of arts at home or with other people outside the course and how this helped their wellbeing. These findings echo those of previous research on arts participation [2,3]. With respect to the *venue*, some students valued the course not being in a mental health service location. All this is consistent with people needing and wanting to develop a life outside or alongside mental health services, as suggested by the recovery models discussed in the introduction [2,7], and consistent with research on arts and other forms of community participation [4,45,46]. Arts venues were appreciated as *inspiring* and as making participants in turn feel special and valued, supporting the suggestion that “cultural provision has to be of a quality to compel attention and sustain engagement” [15] (p. 26).

The theme *positive risk-taking* encapsulated feeling able to move out of one’s comfort zone, with the suggestion from at least one participant (P3) that it was “a nice surprise to actually have a higher expectation...” This is consistent both with Deegan’s suggestion [7] and also evidence [43] that the diagnostic label may reduce expectations of both service user and professional about what can be achieved, and with Boardman and Roberts’ call for positive risk-management [47]. In keeping with previous research [2,3], the theme *positivity of social aspects* seemed closely linked to *positive risk-taking*, in that the positivity seemed to come partly from the initial feeling of anxiety about social interaction and then finding it manageable within the safe space provided. This seems to confirm the importance of employing artists who had skills in participatory work, and involvement of peer trainers. Indeed, a few participants remarked that the *tutors were special*.

Similar to findings in previous research [23], peer trainers reported a sense of personal or professional development, which may afford further opportunities for making personally meaningful contributions. Although the professional artists were not required to have personal experience of mental distress, they too reported a sense of a personal journey, with resonances to Anthony’s definition of recovery [5].

This, and the aforementioned student themes of *learning skills* and experiencing *artistic growth* is also consistent with theorizing about instinctual needs for interest-sharing as a natural human drive [11], which in itself is consistent with Gilbert’s compassionate mind theory, with its three instinctual drives: fight/flight, exploratory, and safeness/soothing [48]. From the perspective of these frameworks perhaps the arts components and sense of relational safety were sufficient for many participants to be able to regulate their fight-flight response and experience exploratory interest-sharing [11], entailing enjoyment, play and learning during their courses: Some then transferred their learning beyond the course itself. Another possible mechanism of arts based activities is the sense of control and agency they may engender, making life feel generally more worthwhile [2]. Research including the present study [2,3] suggests that arts participation supports recovery defined by service users as connectedness, hope, identity, meaning and empowerment [8].

Although some students appreciated the trainers having high expectations of them, for some trainers, modifying (though not necessarily lowering) their expectations seemed necessary in order to fully appreciate what was possible and its value, as in the quote, “This is what it is.” The role of support and supervision, and prior training with artists and peers together, seemed important here. Co-production between artists and peer trainers was reported as sometimes challenging, but this is not unusual, as has been reported about co-production between mental health professionals and service users [49]. When forging a new social belonging cuts across taken-for-granted social groups seen as separate (professional artist, mental health peer), it may entail the need to have some commonalities (such as art experience in peers or distress in artists) accepted and celebrated rather than resisted according to one’s group stereotype. Richards, Holttum and Springham [50] reported that mental health professionals with experience of mental distress found it difficult to reconcile two social identities (patient and professional), apparently because of the social expectations of them being separate groups. Nevertheless, Corrigan and Shapiro’s [51] review of research on what helps dispel stigma concluded that the most effective approach was enabling groups that might stigmatize (or be stigmatized by) the other to come together with a common goal and where members of both groups have equal value.

Although many participants talked about barriers to doing more arts activities, they also talked about doing more, either at home or with others outside the home, or both, consistent with their reports of revitalized creativity and what appeared to be capacity for interest-sharing [11]. Finally, in our exploratory pattern matching [37], self-reported *artistic growth* at the first follow-up predicted participants’ reporting of *improved mental wellbeing* at later follow-ups, and *both positivity of social aspects* and *inspiring venue* were predictive as non-significant trends. The experiences we categorized as *artistic growth* included developing “an artistic personality” (anonymous student feedback), a sense of “freedom” (P10), “creativity” (Sindy, peer trainer), motivation to “take things home” (P3) and emotional expression through arts (P11). While reminiscent of previous research on arts activities [2,3], our follow-up interviews up to nine months post course, and use of pattern matching [37], enabled us to demonstrate a link between these initial experiences and subsequent self-reported mental wellbeing.

### 4.3. Limitations

Our sample size was relatively small and suffered from missing data. It is unknown whether those who could not be contacted for follow-up had worse mental wellbeing or were unavailable for other reasons. The researcher who collected six and nine month follow-up data had also interviewed people at three months and had attended some sessions so that people could make an initial connection with her. This could mean unintentional bias may have influenced her interviewing, such as expecting people who she was aware of gaining a lot from courses early on to report improved wellbeing later. However, it also may have helped her to secure interviews with as many as she did, and made it easier for people to speak freely. The authors tried to guard against bias by having multiple people involved in checking the coding of data and being alert for negative cases. The interviewer had regular opportunity to reflect on potential biases and was careful to ask open questions in the interview as far as possible to avoid leading people to respond in a socially desirable way. In particular, we sought to capture participants’ descriptions of lack of change or worse mental wellbeing in follow-up interviews. No-one reported deterioration but lack of change is well-documented in the results section.

Ideally we would have provided the diagnoses that people had received and the length of time in the mental health system, but we wished to limit the number of questions asked of participants and ask questions more in keeping with an educational than a medical setting. However, the population pool of our sample was the same as for another recent study in the same NHS trust that examined trust records on use of services before and after people attended the full range of recovery college courses rather than only arts based courses [27]: Over 90% of their 194 participants with information about their mental health were classified as “severe and complex” or “psychosis”.

It is highly likely that identified themes were prompted by the topics on the feedback form and in interviews. However, a pragmatic decision was taken to provide structure in both, since previous experience and consultation with service users suggested that participants found it easier to answer questions within a defined framework. Participants also may have wanted to give positive feedback to someone they saw as a member of the team providing the courses. Nevertheless some participants were able to state that the courses made no difference to their wellbeing. Others who felt courses were helpful were more enthusiastic than might be suggested by social-desirability responding alone. In addition, the multiple perspectives from focus groups with the trainers, anonymous student feedback and individual interviews provided good triangulation for themes about the immediate impact of the courses. The mixed-method design also meant that both qualitative and quantitative data indicated improved wellbeing.

The lack of any control or comparison group limits the conclusions that we can draw. All participants were also in usual mental health care, and a few reported engaging in psychological therapy subsequent to their course. This may have explained the mental health improvement of some participants as much or more than the courses. Equally, it is possible that some participants may have felt more able to seek or take up psychological therapy following course attendance.

Given that we were not able to rule out differences in participants’ difficulties before they entered a course, this was not carefully-controlled case-study research [37]. Self-report of improved wellbeing could therefore represent differences in people’s initial mental health or environmental challenges. In order to definitively support the hypotheses that arts based courses facilitate wellbeing due to enabling artistic growth, a positive social experience or enfranchisement projected by a cultural venue, predictions of later wellbeing would either need to be found in a larger sample in a randomized trial with researchers at follow-up blind to trial condition, or in more carefully controlled case-study research that could exclude other possible influences on individual outcome [37].

Finally, the sample sizes for the statistical tests were small, and may have suffered from low power, leading to some Type II errors, especially as we used the conservative two-tailed test. For example, in relation to arts participation the significance level for increase in frequency of participation was 0.097, and there were non-significant trends in the chi-squared tests for a relationship between being classified as an improver and both experiencing positive social aspects of a course, and the venue as inspiring. No outcomes were in a direction opposite to what would be expected if assuming student benefit.

### 4.4. Areas for Further Research

This study has raised some important questions for further research. One is whether our findings of improved mental wellbeing following attendance of arts based recovery college courses would be replicated in a larger, controlled study. Although it can be difficult to follow people up over long periods, it is possible that a larger project with greater resource might put in place additional support for this task so that a higher proportion of participants could be included, both immediately post course and at later follow-up points. This, along with a control group would be important for future research on arts based provision as part of recovery colleges. As well as usual care only, perhaps recovery college courses without an arts base could be the active control. The two standardized measures of wellbeing seemed appropriate in capturing change, and they should ideally be used at follow-up points. In addition a standardized measure of social inclusion would be advisable, although development of such measures is relatively young [52]. In light of this, a measure might be produced on the basis of the qualitative data gathered here, as well as relevant literature in order to build on an existing social inclusion measure, such as that produced by Secker, Hacking, Kent, Shenton and Spandler [53]. In addition, personal recovery might be captured by the use of the innovative ReQol measure [54], which was co-produced with service users.

Our preliminary findings regarding people’s experience during the courses and how that might predict later experience of mental health improvement raise interesting questions for further investigation, to evaluate further the factors that mediate impact of arts based recovery college courses. In particular, experiences we categorized as *artistic growth*, *inspiring venue* and *positivity of social aspects* could be investigated using deductive thematic analysis and pattern matching and a prospective design controlling for severity of difficulties at the outset. This may or may not support a tentative hypothesis from the present study that the experience of liberation, creativity and emotional expression through arts, the connection with others, and the feeling of being valued and part of the wider community, motivate and inspire people to continue arts activities to maintain their mental wellbeing for some time after course attendance. Connection with others and social connection are also present in non-arts activities such as football [45,46], which is also a culturally valued activity for the community served. Together these findings arguably support both O’Neill’s contention about the importance of cultural provision [15] and the exploratory interest-sharing theory [11]. They suggest that cultural provision, whether in the form of arts or other activities and contexts that are valued by people striving to recover a life after mental health diagnosis, may be vital in enabling that transition back to personhood [7,8].

## 5. Conclusions

To our knowledge this is the first in-depth evaluation of recovery college courses that are arts based, and unusually, also involved follow-up interviews at three, six and nine months after the courses. Our findings suggest, tentatively, that the employment of professional artists with experience of participatory work, alongside peer trainers, and holding courses in public venues, adds a quality to the whole experience that may harness a natural human desire for interest-sharing and enable people to do things they previously could not. Although our study has a number of limitations, it does suggest important directions for future research in this area. In any future controlled trials, it would be important to incorporate validated measures of social inclusion, arts participation and personal recovery, and to explore links between participants’ perceptions of the initial course experience and subsequent reports of improved mental wellbeing.

## Figures and Tables

**Table 1:** Outline of arts courses delivered.

Title of Course	Art Form and Approach	Venues
Stories of a Journey—Recovery on Film	Puppetry and animation to tell a recovery story on both an individual and group level	Hawth Theatre, Crawley
Mindful Drama and Storytelling for Supportive Recovery	Drama, storytelling and movement to develop confidence	Ropetackle Arts Centre, Shoreham-by-Sea
Creative Music Making	Music making in a safe environment, involving producing a new piece of music	Worthing Museum, Worthing
Art and Movement for Wellbeing	Art, movement and nature, to build confidence, increase wellbeing and improve health	Clair Hall, Haywards Heath and park and woodland spaces
Recovery and Self Discovery through Arts and Food	Learning new and creative ways to approach food and art: Tasting, making and sharing food and visual art	Towner Gallery, Eastbourne
Creative Singing for Wellbeing	Using voice, breath and posture as tools to deal with anxiety and to produce a voice soundscape	De La Warr Pavilion, Bexhill on Sea
Playing with Print	Self-expression through print making to build confidence in a safe environment	Brighton Museum, Brighton

**Table 2:** Changes in wellbeing from before to after course attendance.

	Mean before (sd)	Mean after (sd)	*T*	*p*
Wellbeing ^1^ (*n* = 14)	2.56 (0.67)	2.89 (0.90)	−2.66	0.020 *
CHOICE ^2^ (*n* = 14)	3.87 (1.83)	5.35 (2.43)	−3.06	0.009 **

^1^ Edinburgh Mental Wellbeing Scale; ^2^ CHoice of Outcome In Cbt for psychosEs Scale; * *p* < 0.05; ** *p* < 0.01.

**Table 3:** Changes in arts participation pre to post course (corrected for Make your Mark (MYM) course attendance).

Activities Reported by *n* = 28 Participants	Median before (Range)	Median after (Range)	*Z*	*p*
Number of different activities (of 13 options) ^1^	3 (0–7)	5 (1–11)	−2.91	0.004 **
Number of different locations (of 8)	2 (0–5)	3 (0–6)	−0.99	0.334
Different people/groups done with (of 6)	1 (0–4)	1 (0–6)	−0.44	0.659
Frequency of activity ^2^	4 (1–5)	4 (1–5)	−1.66	0.097

^1^ Response options; ^2^ Frequency responses from 1 = Once to 5 = Daily; ** *p* < 0.01.

**Table 4:** The theme Transforming students’ lives and its sub-themes.

Sub-Theme	Found in:			
	Students’ Anonymous Feedback	Artist Focus Group	Peer Trainer Focus Group	*n* Participants at Three Month Interview (of 22)
Improved mental wellbeing	Yes	Yes	Yes	16 ^1^
No change in mental health				9 ^1^
Artistic growth	Yes	Yes	Yes	9
Inspiring venue	Yes	Yes	Yes	11
Learning skills	Yes		Yes	7
Positive risk-taking	Yes		Yes	12
Positivity of social aspects	Yes			7
Tutors special				4

^1^ Both themes of *improved mental health* and *no change* were found in data from three participants.

**Table 5:** Sub-themes of the theme ‘Development as trainers’.

Sub-Theme	Found in:	
	Artists	Peer Trainers
Practice development	Yes	Yes
Personal confidence and self-esteem	Yes	Yes
Professional pride	Yes	
Positive risk-taking		Yes

**Table 6:** Components of the main theme ‘Overcoming hurdles’.

Sub-Theme	Found in:		
	Students	Artists	Peer Trainers
Appreciation of support and supervision		Yes	Yes
Concern over attendance		Yes	Yes
Co-production: The good, the bad and the ugly		Yes	Yes
Meeting and not meeting expectations		Yes	Yes
Coping with difference	Yes		Yes

**Table 7:** Participants’ self-report of mental wellbeing at each follow-up.

Participant	Wellbeing at Follow-Up 1	Wellbeing at Follow-Up 2	Wellbeing at Follow-Up 3
**Sustained improvers *n* = 12**			
5	Improved	Improved	Improved
7	Improved	Improved	Improved
10	Improved	Improved	Improved
11	Improved	Improved	Improved
13	Improved	Improved	Improved
18	Improved	Improved	Improved
1	Improved	ND	Improved
2	ND ^1^	Improved	Improved
3	Improved	Improved	ND
14	ND	Improved	Improved
35	Improved & No change	Improved	Improved
36	Improved & No change	Improved	Improved
**Improvers *n* = 5**			
4	Improved	ND	ND
19	Improved	No change	Improved
21	Improved	ND	ND
32	Improved	Improved & No change	Improved
28	No change	Improved	ND
**Possible improvement *n* = 3**			
8	Improved	No change	No change
12	Improved and No change	No change	Improved & No change
44	No change	No change	Improved & No change
**No change *n* = 4**			
6	No change	ND	ND
16	No change	No change	No change
17	No change	No change	No change
34	No change	No change	No change

^1^ ND = No data.

**Table 8:** Quotations illustrating self-reports of improvement and no change.

Participant Number	Participant Earlier Follow-Up	Later Follow-Up
	Participants Reporting Sustained Improvement	
5	*“It’s been a huge impact, definitely. […] I was very desperate before. It’s definitely changed my view on my future.”* (3m) ^1^	*“Since taking that time out for me […] and really just focus my artistic side definitely brought out a happier me again.”* (6m)
7	*“It’s very important to get balance as well. I feel like that’s a big reason why I relapsed [before] because everything was recovery, recovery, recovery and […] no fun.”* (3m)	*“It [MYM] was very important because it was a catalyst in sending me in a new direction […] I met my sponsor through that course so that was incredible.”* (9m) ^2^
10	*“Less social anxiety now. […] I would say I’m almost fully recovered now.”* (6m)	*“I’m nearly recovered now, I guess. […] It helped build a bit of social confidence.”* (9m)
11	*“It undoubtedly lifted my mood at the time and led me on to do other things. It’s helped me with a sense of feeling more confidence to do things.”* (3m)	*“I’ve become sort of generally more confident that I can do things, more confident in myself […]. The overall picture is definitely on the up for me*. *”* (9m)
18	*“I am slightly progressing and getting better I think. You just got to keep thinking that.”* (6m)	*“I’m better than I was. […] Probably got a bit of confidence from the course and moved that confidence over to playing [musical instrument] down the pub.”* (9m)
**Participants reporting improvement**		
32	*“But mentally I feel quite optimistic and more hopeful.”* (6m) ^3^	*“My mood has greatly improved since attending the creative singing for wellbeing course. I have felt confident for longer periods of time.”* (9m)
28	*“It’s been alright over the time […]. I haven’t had any sort of upsets since the course.”* (3m)	*“In terms of wellbeing, it’s pretty good really.”* (6m)
**Participants reporting possible improvement**		
8	*“I think it has made me a bit more optimistic and hopeful—the experience, memories of doing it.”* (3m)	Interviewer: *“How has your mood been?“* P8: *“Well that’s really bad when I’m not well. […] When I feel physically better, I feel mentally better.”* (9m) ^4^
**Participants reporting no change**		
16	*“Well yeah, you know, about the same really. It’s just not a lot of difference. Each day’s the same for me really.”* (6m)	Interviewer: *“Do you feel the course helped with your mood?“* P16: *“I don’t think it makes any difference really—circumstances.”* (9m)
34	Interviewer: *“How’s your mood been?“* P34: *“Down […].When I take my tablets it calms me down for a bit.”* (6m)	*“Since I signed on the courses, my mood is better. Since I finished the courses my mood has been down.”* (9m)

^1^ 3m = three month follow-up. ^2^ Participant 7 reported improvement in dealing with diabetes and alcohol addiction at 6m; ^3^ P32 reported a mental health admission of a few days; ^4^ P8 has a fluctuating physical condition.

**Table 9:** Themes and sub-themes at each follow-up interview (*n* = 24)

Main Theme	Sub-Themes	*n* Participants at Three Months	*n* at Six Months	*n* at Nine Months
Maintaining wellbeing	Using skills learned	7	6	5
	Another course (not MYM)	5	3	3
	Job or voluntary work	1	3	1
	Increased social inclusion	6	14	10

**Table 10:** Number of participants doing more arts or finding barriers to it at follow-up.

Arts activities Main Theme	Arts Activities Sub-Themes	*n* Participants at Six Months	*n* at Nine Months
Doing more arts	Doing more arts	11	7
	Continued or arts rekindled	3	6
Barriers to doing more arts	Competing demands	7	5
	No opportunity	4	3
	Physical health	3	1
	Mental health	4	2

**Table 11:** Variables (as self-reported) associated with more and less self-reported improvement in mental wellbeing.

	Improvers and Sustained Improvers ^1^ *n* = 15	Possible Improvers & No Changers ^2^ *n* = 6	*P (Fisher’s Exact)*
**Perceptions of RC at FU1**	***n* (%)**	***n* (%)**	
Artistic growth	9 (60)	0 (0)	0.019 *
Positive risk-taking	10 (67)	2 (33)	0.331
Positivity of social aspects	7 (41)	0 (0)	0.061 ^3^
Inspiring venue	10 (67)	1 (17)	0.063 ^3^
Tutors special	3 (20)	0 (0)	0.526
**Prior factors reported at FU1**			
Previous art affinity (liking or interrupted arts career)	6 (40)	2 (33)	1.00
Little prior arts engagement	0 (0)	1 (17)	0.268

^1^ Reported improvement in mental wellbeing at six and nine month follow-up, or reported improvement at latest follow-up with data; ^2^ Reported no change in wellbeing on both six and nine month follow-ups; ^3^ Non-significant trend; * *p* < 0.05.

## Supplementary Materials

The following are available online at http://www.mdpi.com/1660-4601/15/6/1170/s1, Figure S1: Clinical Governance Certificate of Approval, Table S1: Scores on anonymous post course feedback forms, Table S2: Semi-structured interviews with service users at three month follow-up—October 2016, Table S3: Semi-structured interviews with service users at six month follow-up—January 2017, Table S4: Semi-structured interviews with service users at nine month follow-up—April 2017.
