# Supplementary material for: Evaluation of Arts based Courses within a UK Recovery College for People with Mental Health Challenges
Source: Int J Environ Res Public Health. 2018 Jun 4;15(6):1170. doi: 10.3390/ijerph15061170 (PMC6025642; doi:10.3390/ijerph15061170)
Supplement: Supplementary file 1 [file ijerph-15-01170-s001.zip › Zip file/Figure S1_Clinical_Governance Certificate.pdf]

Sussex Partnership NHS
NHS Foundation Trust

This is to certify that

An evaluation of participant experiences during and after attendance on Arts based Recovery College courses investigating participants self-report about any changes in arts participation and mental health service use.

Has gained governance approval from the

Clinical Audit Team

On 28 April 2016

Wendy Harlow,
Head of Clinical Audit
