# Supplementary material for: Evaluation of Arts based Courses within a UK Recovery College for People with Mental Health Challenges
Source: Int J Environ Res Public Health. 2018 Jun 4;15(6):1170. doi: 10.3390/ijerph15061170 (PMC6025642; doi:10.3390/ijerph15061170)
Supplement: Supplementary file 1 [file ijerph-15-01170-s001.zip › Zip file/Table S1.docx]

**Table S1**: Scores on anonymous post course feedback forms

| **Feedback form statements** | **N^1^** | **Median** | **Min.** | **Max.** | **% positive** | **% middle score** | **% negative** |
| --- | --- | --- | --- | --- | --- | --- | --- |
| Helpful and informative | 16 | 1^2^ | 1 | 5 | 94 | 0 | 6 |
| Recommend to family and friends | 25 | 1 | 1 | 5 | 92 | 4 | 4 |
| Has helped my recovery | 25 | 2 | 1 | 4 | 76 | 16 | 8 |
| Increased knowledge and skills | 25 | 1 | 1 | 4 | 88 | 8 | 4 |
| Helpful to have a peer trainer | 15 | 1 | 1 | 5 | 87 | 7 | 7 |
| Tutors helpful and accessible | 24 | 3 | 1 | 3 | 38 | 63 | 0 |
| Other students helped me learn | 24 | 3 | 1 | 3 | 42 | 58 | 0 |
| Quality of materials | 15 | 3 | 3 | 3 | 0 | 100 | 0 |
| Quality of Venue | 23 | 3 | 1 | 3 | 30 | 70 | 0 |

^1^ Participants did not rate every item ^2^ Score range 1 = Strongly agree to 5 = Strongly disagree
