# Supplementary material for: Evaluation of Arts based Courses within a UK Recovery College for People with Mental Health Challenges
Source: Int J Environ Res Public Health. 2018 Jun 4;15(6):1170. doi: 10.3390/ijerph15061170 (PMC6025642; doi:10.3390/ijerph15061170)
Supplement: Supplementary file 1 [file ijerph-15-01170-s001.zip › Zip file/Table S2.docx]

**Table S2:** Semi-structured interviews with service users at three month follow-up – October 2016

| **Main questions** | **Supplementary questions** |
| --- | --- |
| 1. What did you think of the course? | 1. How did you feel about the course? |
|  | 1. What did you enjoy? |
|  | 1. What didn’t you like/ what was a problem for you? |
| 1. What did you think of the venue? | 1. Did it influence your enjoyment of the course? |
|  | 1. What was the impact at the time? Since? |
|  | 1. Has being at that venue changed your view of the accessibility of the arts? |
| 1. How have things been since the course? | 1. Have you been doing any arts-based activities since the course finished? |
|  | 1. Is this a new activity since the course? |
|  | 1. Do you think your view of the arts has changed since the course? |
|  | 1. What about social activities? |
|  | 1. Is this a new activity since the course? |
| 1. Have you been involved in mental health services in the past? | 1. Is that how you came to be on the course? |
| 1. Do you feel there’s been any change in your mental health (mood) since the course? | 1. Has there been a change in your involvement in mental health services since the course? |
|  | 1. What do you think that change is down to? |
| 1. Anything else/ final thoughts? |  |
