# Supplementary material for: Evaluation of Arts based Courses within a UK Recovery College for People with Mental Health Challenges
Source: Int J Environ Res Public Health. 2018 Jun 4;15(6):1170. doi: 10.3390/ijerph15061170 (PMC6025642; doi:10.3390/ijerph15061170)
Supplement: Supplementary file 1 [file ijerph-15-01170-s001.zip › Zip file/Table S3.docx]

**Table S3:** Semi-structured interviews with service users at six month follow-up – January 2017

| **Main questions** | **Supplementary questions** |
| --- | --- |
| 1. How have you been? | 1. How has your physical health been? |
|  | 1. How has your mental health been? |
|  | 1. How have things been socially? |
| 1. Has there been any change since last time we talked? | 1. Has Christmas impacted these things? |
| 1. How has your arts participation been? | 1. Has there been a change since we last talked? |
| 1. Anything else/ final thoughts? |  |
