# Supplementary material for: Evaluation of Arts based Courses within a UK Recovery College for People with Mental Health Challenges
Source: Int J Environ Res Public Health. 2018 Jun 4;15(6):1170. doi: 10.3390/ijerph15061170 (PMC6025642; doi:10.3390/ijerph15061170)
Supplement: Supplementary file 1 [file ijerph-15-01170-s001.zip › Zip file/Table S4.docx]

**Table S4:** Semi-structured interviews with service users at nine month follow-up – April 2017

| **Main questions** | **Supplementary questions** |
| --- | --- |
| 1. How have you been? | 1. How has your physical health been? |
|  | 1. How has your mental health been? |
|  | 1. How have things been socially? |
|  | 1. Have there been any change since last time we talked? |
| 1. How has your arts participation been? | 1. Has there been a change since we last talked? |
| 1. Anything else/ final thoughts? |  |
